# Improved In-Flight Estimation of Inertial Biases through CDGNSS/Vision Based Cooperative Navigation

**DOI:** 10.3390/s21103438

**Published:** 2021-05-14

**Authors:** Flavia Causa, Giancarmine Fasano

**Affiliations:** Department of Industrial Engineering, University of Naples Federico II, Piazzale Tecchio 80, 80125 Naples, Italy; giancarmine.fasano@unina.it

**Keywords:** cooperative navigation, extended Kalman filter, dynamic inertial bias estimation, relative motion geometry, visual tracking, carrier-phase differential GNSS

## Abstract

This paper discusses the exploitation of a cooperative navigation strategy for improved in-flight estimation of inertial sensors biases on board unmanned aerial vehicles. The proposed multi-vehicle technique is conceived for a “chief” Unmanned Aerial Vehicle (UAV) and relies on one or more deputy aircrafts equipped with Global Navigation Satellite System (GNSS) antennas for differential positioning which also act as features for visual tracking. Combining carrier-phase differential GNSS and visual estimates, it is possible to retrieve accurate inertial-independent attitude information, thus potentially enabling improved bias estimation. Camera and carrier-phase differential GNSS measurements are integrated within a 15 states extended Kalman filter. Exploiting an ad hoc developed numerical environment, the paper analyzes the performance of the cooperative approach for inertial biases estimation as a function of number of deputies, formation geometry and distances, and absolute and relative dynamics. It is shown that exploiting two deputies it is possible to improve biases estimation, while a single deputy can be effective if changes of relative geometry and dynamics are also considered. Experimental proofs of concept based on two multi-rotors flying in formation are presented and discussed. The proposed framework is applicable beyond the domain of small UAVs.

## 1. Introduction

Nowadays, Unmanned Aerial Vehicles (UAVs) represent a popular solution for executing tasks in several markets and applications [1], such as delivery of goods [2], surveillance and monitoring [3], inspection and mapping [4], precision agriculture [5], and cinematography [6]. The usage of flying platforms allows reducing time and cost of the mission, while guaranteeing high flexibility. This improves mission performance and/or enables missions which were not feasible at all. However, capability of UAVs to carry out their mission while autonomously or remotely piloted, strictly depends on their navigation performance which may require to be very accurate (at least in post processing stage) in several applications, such as mapping and photogrammetry.

UAV navigation is usually tackled by fusing inertial and GNSS (global navigation satellite system) measurements, which for their complementary properties are usually combined in Kalman filters (KF). Inertial measurements consist of three axes gyroscopes’ and accelerometers’ observables, retrieved with an inertial measurement unit (IMU). These measurements are affected by different error sources including a time-varying in-run bias for each channel, which if not correctly estimated, can spoil the performance in positioning, velocity, and attitude estimate. Residual uncompensated inertial biases may also play a key role in the positioning error growth rate in absence of reliable GNSS coverage.

The problem of in-run bias estimation has been widely tackled in the open literature. It requires combining gyros and accelerometers measurements with additional information which could be either measurements provided by other sensor sources, or information on the actual IMU/platform configuration. Several strategies have been developed in the last few years which include static and dynamic inertial biases calibration.

Static calibration is the most used technique, since the assumption of static configuration is effective in improving the bias estimation process. It requires the platform to be steady for a time interval that can last several minutes (which can pose challenges in some application scenarios). The zero velocity upload (ZUPT) [7,8] is the most used static technique. It uses, as an additional measurement within the navigation filter, the assumption that the linear velocity is zero. This assumption allows correctly estimating the vertical accelerometer bias and the x and y gyro biases [9,10]. Reference [11] studied the ZUPT problem, demonstrating that in a 15 variables state (3 positions, 3 velocities, 3 attitude angles, 3 accelerometers’, and 3 gyroscopes’ biases), 6 out of 15 variables are unobservable. It also defined subspaces of individually observable errors, coupling the variables which are dependent from each other.

To have a full estimate of all the accelerometer and gyro biases, a common solution is to place the IMU sensor in a set of static positions (attitudes) to solve the full in-run bias estimation problem [12,13,14]. This requires a longer estimation time for in-run bias estimation than the ZUPT solution. Piecewise static position bias calibration concept has been recently extended by [15], which creates a solution that exploits IMU rotation along sensitive axes. Indeed, IMU calibration may benefit from platform maneuvers which improve bias observability.

Due to this property, several studies deal with dynamic bias calibration, exploiting several external sensors as reference for bias estimation. In addition, due to bias instability, depending on mission duration, in-flight bias estimation may be needed also in the case of accurate initialization. Kalman filters or non linear observers, which guarantee global convergence [16] have been used for fusing IMU information with other sources of measurements. Several authors integrate IMU measurements either with GNSS [10,17] or odometry information [9,16] to improve biases estimation, and provide biases observability analyses based on platform motion. Partial IMU bias observers (gyroscope only) have been developed, accounting only for gyro measurements [18], or for full IMU measurements sets [19,20,21], where GNSS information has also been accounted as positioning reference. Reference [22] estimates position, velocity, attitude, and gyro biases by fusing IMU measurements with altimeter, heading estimate, and line-of-sight (LOS) estimates given by a camera. A full filter state with 15 variables is used in [23] for retrieving the full pose of the platform with a camera, demonstrating that the 15 variables set is fully observable when both position and attitude estimates are available. Therefore, in-run bias estimation and IMU calibration can be performed, integrating IMU outputs with GNSS measurements which provides a position reference, under dynamic and static configurations if IMU-independent attitude information is available.

The authors proposed in [24,25] an attitude estimation strategy exploiting cooperative navigation [26], which uses unit vectors estimated in geographic and body frame. One or two vehicles, named deputies, have been used for improving the attitude accuracy of a “chief” vehicle and LOS vector in the local frame and body frame have been retrieved with carrier-phase differential GNSS (CDGNSS) and visual-based information, respectively. This information has been used to retrieve an estimate of the chief attitude which is independent from inertial (IMU) and magnetic measurements. A similar approach, consisting of using LOS measurements only, has been used in [27], demonstrating that at least three LOS directions are needed to provide the full observability of the 15 variables state.

Cooperative or networked navigation is intended as the operation of using networked relative measurements (e.g., range-based, angle-based) to the aim of the navigation performance increase [28]. This approach has been widely used in the open literature with promising outcomes especially when navigating under non-nominal GNSS coverage [29,30]. As an example, a networked system of the GNSS ground receiver has been exploited in [31,32]. In the cooperative navigation framework, magnetometer bias calibration has been carried out in [33], but no comprehensive studies concerning the potential and applicability of the concept for in-flight inertial biases estimation have been proposed in the open literature, to the best of the authors’ knowledge.

This paper analyzes the potential of the cooperative strategies introduced in [24,25] towards in-flight estimation of inertial biases, using extensive numerical analyses and experimental results. Compared with recent literature, the main innovative points are:

- Using CDGNSS and LOS measurements makes the bias estimation technique independent from the accurate knowledge of the cooperative targets’ absolute position, unlike [27]. This reduces the set-up time required to precisely place the targets and estimate their absolute position, and makes the proposed solution independent from the ground infrastructure and more adaptive for being applied to different environments. In addition, retrieving positioning from GNSS measurements allows using cooperative vehicles only for attitude estimate, which can reduce the number of minimum required targets, from 3 [27] to 2.
- Differently from other solutions in literature [23,27], the proposed approach uses moving targets whose trajectories can be planned and executed so that they always lie in the chief camera field of view (FOV) [34], thus avoiding the need of a large set of targets.
- The proposed method offers an innovative instrument to perform calibration during the flight which can be required in case of relatively long flights, and/or when more accurate observables are required during a specific segment of the flight.
- Despite full observability guaranteed by the usage of two deputy vehicles [25], the paper investigates bias estimation performance with a single deputy combining platform motion [10,17] with external cooperative aiding [23,24,27].

The paper is organized as follows. Section 2 introduces the cooperative navigation strategy. Section 3 introduces the nomenclature used in the paper. Navigation state estimation and its equations are detailed in Section 4. A numerical analysis of bias estimation performance is presented in Section 5, while Section 6 presents experimental results from flight experiment with multi-rotors. Finally, Section 7 draws the conclusion of the present work.

## 2. Cooperative Navigation Strategy for Inertial Biases Estimation

**Figure 1:**
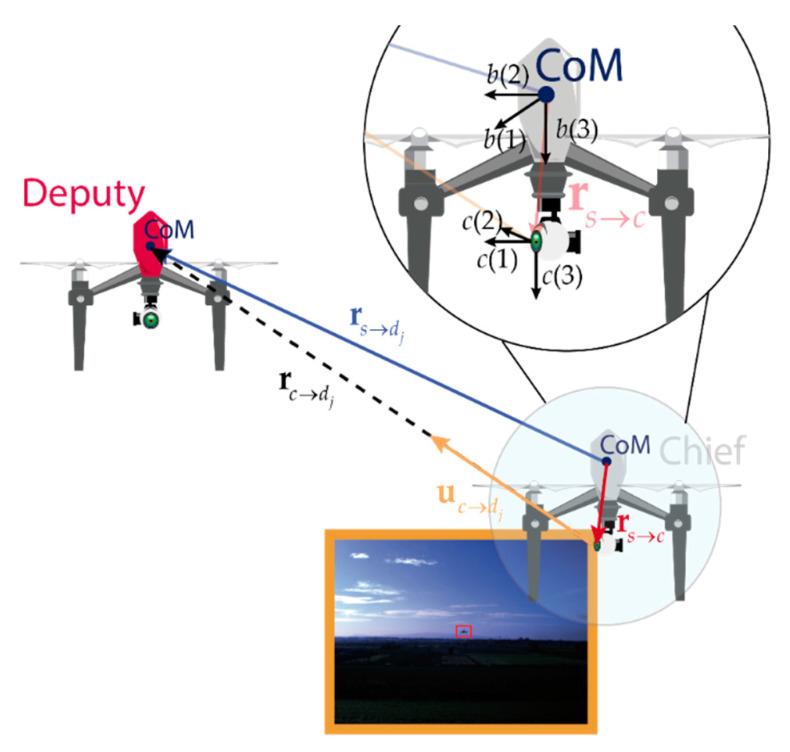
Cooperative navigation concept. Blue quantities are measured by CDGNSS techniques, orange quantities are estimated with cameras.

This paper uses the cooperative navigation strategy firstly introduced in [35] and then extended in [24,25]. Partial (in case of a single deputy) or full (in case of two or more deputies) attitude information is provided by estimating reference directions in two reference frames, i.e., the local frame defined as north east down (NED) frame and the body reference frame (BRF) for the specific case of UAV navigation. The LOS direction in BRF is estimated with a camera and a visual-tracking algorithm, able to track the deputy UAV(s) during the flight. Relative azimuth and elevation are converted from camera reference frame (CRF) to BRF with the known relative orientation of these two frames, which can be obtained via off-line extrinsic calibration in strapdown camera installation. On the other hand, a very accurate estimate of the baseline between the two vehicles in NED is achievable under nominal GNSS coverage, using CDGNSS techniques. For the sake of clarity, a conceptual image of the used framework with a single deputy is reported in Figure 1, and a visual interpretation of the quantities measured by CDGNSS and visual processing are reported in blue (CDGNSS baseline) and in orange (LOS unit vector retrieved from camera), respectively. The cooperative measurement reported in this manuscript is referred to as CDGNSS/vision measurement. Detailed information about the quantities reported in the figure and their usage within the chief navigation architecture are reported in Section 3 and Section 4.

## 3. Nomenclature

Before analyzing the details of the algorithm for state estimation and its equations, this section is in charge of defining the notation that will be used along in the manuscript. Bold, **a**, and italic, *a*, variables indicate respectively vector and scalar quantities. The projection of a vector **a** in the reference frame *i*, is indicated with **a***^i^*. This paper uses NED, chief’s BRF, and CRF which are respectively indicated with the superscripts *n*, *b*, and *c*. Chief BRF is centered in its center of mass (CoM), whereas CRF is centered at camera location. See Figure 1, where camera and body frame axes are reported for the chief vehicle, assuming *a*(*l*) is the *l*-th axis of the frame *a*.

The three components of the vector **a***^i^* in the frame *i* are expressed as $\left[\begin{array}{ccc} a^{i}(1) & a^{i}(2) & a^{i}(3) \end{array}\right]$. To simplify the notation when the NED frame is accounted for, the axis indices reported within the brackets are usually replaced with the letters *n*, *e*, and *d* to indicate the north east and down direction, respectively. Therefore $a^{n}=\left[\begin{array}{ccc} a^{n}(n) & a^{n}(e) & a^{n}(d) \end{array}\right]$. A matrix *A*, is reported in capital style. $C_{i}^{j}$ indicates the rotation matrix which allows transforming a vector from the frame *i* to the frame *j*, such as $a^{j}=C_{i}^{j}a^{i}$.

The position can be represented with two conventions: **p** indicates the position expressed as geographic coordinates (latitude *l*, longitude *λ*, and altitude *h*) and $r_{a\to b}$ indicates the vector going from location *a* to location *b*, which unit vector is indicated with $u_{a\to b}$. Referring to Figure 1, the center of mass location of the chief and the *j*-th deputy have been indicated with *s* and *d_j_*, respectively. The vector measuring their distance which is given by the CDGNSS processing is indicated with $r_{s\to d_{j}}$. Chief’s camera location is indicated with *c*, and the vector connecting camera location with the deputy CoM is $r_{c\to d_{j}}$, whose associated unit vector $u_{c\to d_{j}}$ is measured by the camera. $r_{s\to c}$ is the distance between the chief’s center of mass and origin of the chief’s camera frame.

The derivative of a scalar *a* with respect to a vector **v***^i^* is a 1 × 3 matrix indicated with $\partial a/\partial v^{i}$, whose *l*-th component is the derivative of the scalar *a*, with respect to the *l*-th component of the vector **v***^i^*. Conversely, the 3 × 3 matrix indicating the derivative of a vector **q***^i^* with respect to a vector **v***^i^* is indicated with $\partial q^{i}/\partial v^{i}$, and the element at the *l*-th raw and *j*-th column is $\partial q^{i}\left(l\right)/\partial v^{i}\left(j\right)$.

## 4. Cooperative Navigation Filter

**Figure 2:**
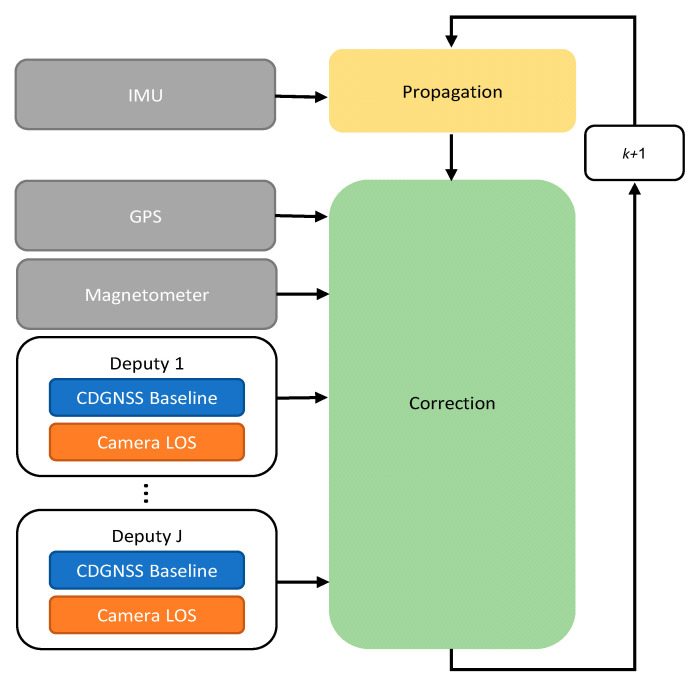
Cooperative navigation filter. Non cooperative measurements are depicted in gray. For each deputy CDGNSS and vision, measurements are depicted respectively in blue and orange.

The navigation architecture used for estimating the state of the chief vehicle is represented in Figure 2. It is based on the extended Kalman filter (EKF) described in [36], and assumes the vehicle state is composed by position **p** in geographic coordinates, velocity expressed in NED frame **v***^n^*, attitude from NED to BRF parametrized by heading *ψ*, pitch *θ* and roll *φ* angles (defined with a 3-2-1 rotation sequence of Euler angles), and the 3 × 1 vectors including the accelerometer and gyroscope biases, expressed in BRF, i.e., $b_{a}^{b}$ and $b_{g}^{b}$, respectively. The filter propagates and corrects the state’s error *δ***x**, which is given by:

(1) $$\delta x=\left[\begin{array}{c} \delta p\\ \delta v^{n}\\ \rho \\ \delta b_{a}^{b}\\ \delta b_{g}^{b} \end{array}\right];\begin{array}{c} \delta p={\left[\begin{array}{ccc} \delta l & \delta \lambda & \delta h \end{array}\right]}^{T}\\ \delta v^{n}={\left[\begin{array}{ccc} \delta v^{n}(n) & \delta v^{n}(e) & \delta v^{n}(d) \end{array}\right]}^{T}\\ \rho ={\left[\begin{array}{ccc} \rho (n) & \rho (e) & \rho (d) \end{array}\right]}^{T} \end{array},$$

where **ρ** represents the attitude error vector expressed in the NED reference frame, as reported in [36]. Its components are indicated with *ρ*.

The filter propagates the state and its error with the widely known inertial navigation equations, which uses IMU measurements to predict the UAV state and its error at the current step *k*, starting from their best estimate at the previous step *k*-1. The WGS84 model has been used for predicting the local gravity vector [7], to have a more accurate estimate of the down component of the accelerometer bias, especially when experimental data are used. Inertial propagation equations are not reported here for the sake of brevity. The interested reader is referred to [36] for further details.

Correction steps use both cooperative and uncooperative measurements (reported in gray in Figure 2). Non cooperative measurements consist of the magnetometer and GNSS outputs, which are complemented with cooperative measurements coming from several (*J*) deputies.

For each deputy, the cooperative measurement to be used for attitude estimate is given by combining CDGNSS and visual output which are reported in blue and orange in Figure 2, respectively.

Correction equation expresses the state error as a function of the measurement residual *δ***y**, through the measurement matrix *H*. It can be written as:

(2) δy=Hδx+w.

where **w** is the measurement noise associated with the residual, whose covariance matrix is *R*.

Equation (2) can be rewritten for the specific filter reported in Figure 2, as:

(3) $$\left[\begin{array}{c} \delta y_{GNSS}\\ \delta y_{MAG}\\ \delta y_{1}\\ \vdots \\ \delta y_{J} \end{array}\right]=\left[\begin{array}{ccccc} H_{GNSS,p} & 0_{m\times 3} & 0_{m\times 3} & 0_{m\times 3} & 0_{m\times 3}\\ 0_{1\times 3} & 0_{1\times 3} & H_{MAG,\rho } & 0_{1\times 3} & 0_{1\times 3}\\ & & H_{1} & & \\ & & \vdots & & \\ & & H_{J} & & \end{array}\right]\left[\begin{array}{c} \delta p\\ \delta v^{n}\\ \rho \\ \delta b_{a}^{b}\\ \delta b_{g}^{b} \end{array}\right]\;R=\left[\begin{array}{ccccc} R_{GNSS} & 0_{m\times 1} & 0_{m\times 2} & \cdots & 0_{m\times 2}\\ 0_{1\times m} & R_{MAG} & 0_{1\times 2} & \cdots & 0_{1\times 2}\\ 0_{2\times m} & 0_{2\times 1} & R_{1} & \cdots & 0_{2\times 2}\\ \vdots & \vdots & \vdots & \ddots & \vdots \\ 0_{2\times m} & 0_{2\times 1} & 0_{2\times 2} & \cdots & R_{J} \end{array}\right]$$

where *H_a,_***_b_** is the matrix that connects the measurement *a* with the part **b** of the state which could be position **p**, velocity **v**, and attitude **ρ**. *R_a_* is the covariance matrix associated to the measurement *a*. 0*_a_*_×*b*_ indicates a matrix composed by all zero elements with *a* rows and *b* columns. *δ***y***_GNSS_* and *δy_MAG_* are the GNSS and magnetometer residuals. *δ***y***_j_* is the residual associated to the cooperative measurement related to the *j*-th deputy, with *j* = 1, …, *J*, and *H_j_* and *R_j_* are their associated measurement and covariance matrices. Detailed derivation of *δ***y***_j_*, *H_j_*, and *R_j_* are reported in Section 4.1.

GNSS pseudorange measurements are tightly integrated within the Kalman filter and the GNSS residual number (*m*) depends on the number of available satellites. Pseudorange measurements only depend on the chief position. Therefore, GNSS residual only combines with position error.

Magnetometer residual is a scalar residual on the heading angle, which is coupled only with the attitude part of the state. For the sake of brevity, details about magnetometer and GNSS residual and covariance matrices are omitted from this manuscript. For further details, the interested reader is referred to [29].

### 4.1. Cooperative Measurement Equation

From Equation (2), the measurement equation for the *j*-th deputy can be written as $\delta y_{j}=H_{j}\delta x+w_{j}$. Where **w***_j_* is a Gaussian noise with covariance *R_j_*. This section is in charge of deriving the terms composing the measurement equation for the cooperative contribution of the *j*-th deputy. Detailed derivation of *δ***y***_j_*, *H_j_*, and *R_j_* is presented hereafter.

The measured distance between the camera and deputy’s center of mass measured in NED, i.e., $r_{c\to d_{j}}^{n}$, can be converted to the LOS direction in CRF thanks to the following formula:

(4) $$u_{c\to d_{j}}^{c}=C_{b}^{c}C_{n}^{b}\frac{r_{c\to d_{j}}^{n}}{\left|r_{c\to d_{j}}^{n}\right|}=C_{b}^{c}C_{n}^{b}u_{c\to d_{j}}^{n},$$

where|**a**| is the operator yielding the norm of the vector in the brackets. Observing from Figure 1, that $r_{c\to d_{j}}^{n}=r_{s\to d_{j}}^{n}-r_{s\to c}^{n}=r_{s\to d_{j}}^{n}-C_{b}^{n}r_{s\to c}^{b}$, and assuming $r_{s\to c}^{}$ negligible in with respect to $r_{s\to d_{j}}^{}$ when computing the norm, Equation (4) can be rewritten as:

(5) $$u_{c\to d_{j}}^{c}\approx C_{b}^{c}C_{n}^{b}\frac{r_{s\to d_{j}}^{n}-C_{b}^{n}r_{s\to c}^{b}}{\left|r_{s\to d_{j}}^{n}\right|}=C_{b}^{c}C_{n}^{b}u_{s\to d_{j}}^{n}-C_{b}^{c}\frac{r_{s\to c}^{b}}{\left|r_{s\to d_{j}}^{n}\right|}.$$

Indicating with [a×] the skew symmetric matrix associated with vector **a**, and with $\hat{a}$ the predicted quantity and δa the error associated to that quantity so that the true value is $a=\delta a+\hat{a}$, Equation (5) becomes:

(6) $${\hat{u}}_{c\to d_{j}}^{c}+\delta u_{c\to d_{j}}^{c}=C_{b}^{c}{\hat{C}}_{n}^{b}\left(I-\left[\rho \times \right]\right)\left({\hat{u}}_{s\to d_{j}}^{n}+\delta u_{s\to d_{j}}^{n}\right)-C_{b}^{c}\frac{r_{s\to c}^{b}}{\left|{\hat{r}}_{s\to d_{j}}^{n}\right|}.$$

The BRF to CRF rotation matrix is assumed to be perfectly known, therefore the estimated parameters of camera calibration $C_{b}^{c}$ and $r_{s\to c}^{b}$ are assumed to be known without errors. Rearranging Equation (6), so to find the CDGNSS/vision residual, i.e., Δ**u***^c^*, yields:

(7) $$\Delta u^{c}={\hat{u}}_{c\to d_{j}}^{c}-C_{b}^{c}{\hat{C}}_{n}^{b}{\hat{u}}_{s\to d_{j}}^{n}+C_{b}^{c}\frac{r_{s\to c}^{b}}{\left|{\hat{r}}_{s\to d_{j}}^{n}\right|}=C_{b}^{c}{\hat{C}}_{n}^{b}\left[{\hat{u}}_{s\to d_{j}}^{n}\times \right]\rho +C_{b}^{c}{\hat{C}}_{n}^{b}\delta u_{s\to d_{j}}^{n}-\delta u_{c\to d_{j}}^{c}.$$

The CDGNSS/vision residual is a 3 × 1 vector, which includes two unit vectors estimated in CRF and NED. The so obtained quantity has one component dependent on the other two. To avoid dealing with linear dependent measurements, which makes the associated covariance matrix rank-deficient, i.e., not invertible, a linear independent measurement vector can be obtained converting the unit vector in Equation (7) in angular residuals, i.e., Azimuth *Az* and elevation *El* residuals, so that:

(8) $$\Delta \xi_{j}={\xi \left({\hat{u}}_{c\to d_{j}}^{c}\right)|}_{cam}-{\xi \left(C_{b}^{c}{\hat{C}}_{n}^{b}{\hat{u}}_{s\to d_{j}}^{n}-C_{b}^{c}\frac{r_{s\to c}^{b}}{\left|{\hat{r}}_{s\to d_{j}}^{n}\right|}\right)|}_{CDGNSS}=\frac{\partial \xi }{\partial u_{c\to d_{j}}^{c}}C_{b}^{c}{\hat{C}}_{n}^{b}\left[{\hat{u}}_{s\to d_{j}}^{n}\times \right]\rho +\frac{\partial \xi }{\partial u_{c\to d_{j}}^{c}}C_{b}^{c}{\hat{C}}_{n}^{b}\delta u_{s\to d_{j}}^{n}-\frac{\partial \xi }{\partial u_{c\to d_{j}}^{c}}\delta u_{c\to d_{j}}^{c}.$$

where ξ could be either *Az* or *El* angle estimated starting from a unit vector. As an example, considering a generic unit vector expressed in CRF $u^{c}$, *Az* and *El* are:

(9) $$\begin{array}{cc} Az\left(u_{}^{c}\right)=\tan^{-1}\left(\frac{u^{c}(2)}{u^{c}(1)}\right); & El\left(u_{}^{c}\right)=-\sin^{-1}\left(u_{}^{c}(3)\right) \end{array},$$

where $\partial \xi /\partial u^{c}$ represents the derivative of the angle ξ with respect to $u^{c}$.

To highlight the source of measurement residual, the subscript *cam* and *CDGNSS* have been reported in Equation (8). $\xi \left({\hat{u}}_{c\to d_{j}}^{c}\right)$ can be obtained directly by converting the pixel information of the deputy in the chief’s camera frame, using pinhole camera model. Whereas, $\xi \left(C_{b}^{c}{\hat{C}}_{n}^{b}{\hat{u}}_{s\to d_{j}}^{n}-C_{b}^{c}r_{s\to c}^{b}/\left|{\hat{r}}_{s\to d_{j}}^{n}\right|\right)$ is obtained starting from the CDGNSS measured baseline, i.e., $\left({\hat{r}}_{s\to d_{j}}^{n}\right)$, its associated unit vector $\left({\hat{u}}_{s\to d_{j}}^{n}\right)$ and the knowledge of the camera position with respect to BRF $\left(r_{s\to c}^{b}\right)$.

The errors $\delta u_{s\to d_{j}}^{n}$ and $\delta u_{c\to d_{j}}^{c}$ are associated respectively to CDGNSS and visual estimate. Converting $\delta u_{s\to d_{j}}^{n}$ as a function of $\delta r_{s\to d_{j}}^{n}$ and expressing $\delta u_{c\to d_{j}}^{c}$ in terms of camera angular error (*δ_cam_*), the right side of Equation (8) becomes:

(10) $$\Delta \xi_{j}=\frac{\partial \xi }{\partial u_{c\to d_{j}}^{c}}C_{b}^{c}{\hat{C}}_{n}^{b}\left[{\hat{u}}_{s\to d_{j}}^{n}\times \right]\rho +\frac{\partial \xi }{\partial u_{c\to d_{j}}^{c}}C_{b}^{c}{\hat{C}}_{n}^{b}\frac{\partial u_{s\to d_{j}}^{n}}{\partial r_{s\to d_{j}}^{n}}\delta r_{s\to d_{j}}^{n}-\frac{\partial \xi }{\partial u_{c\to d_{j}}^{c}}\frac{\partial u_{c\to d_{j}}^{c}}{\partial \xi }\delta_{cam}.$$

$\delta r_{s\to d_{j}}^{n}$ and *δ_cam_* are respectively CDGNSS and camera error. The first represents the vector including the error along each component of the baseline estimated with the CDGNSS technique. Its standard deviation (STD) components in NED frame are *σ_CDGNSS_*(*n*), *σ_CDGNSS_*(*e*), and *σ_CDGNSS_*(*d*). On the other hand, *δ_cam_* is the error in camera identification of the target which coincides with the instantaneous field of view (IFOV) and has as STD *σ_cam_*. Equation (10) is the measurement equation for the CDGNSS/vision observable. *δ***y***_j_*, *H_j_*, and *R_j_* can be extracted from this equation, considering the left side of the equation for the measurement residual, and the state dependent and state independent part of the right side of the equation for measurement and covariance matrix, respectively. Therefore:

(11) $$\delta y_{j}=\left[\begin{array}{c} Az_{j}\\ El_{j} \end{array}\right]=\left[\begin{array}{c} {Az\left({\hat{u}}_{c\to d_{j}}^{c}\right)|}_{cam}-{Az\left(C_{b}^{c}{\hat{C}}_{n}^{b}{\hat{u}}_{s\to d_{j}}^{n}-C_{b}^{c}\frac{r_{s\to c}^{b}}{\left|{\hat{r}}_{s\to d_{j}}^{n}\right|}\right)|}_{CDGNSS}\\ {El\left({\hat{u}}_{c\to d_{j}}^{c}\right)|}_{cam}-{El\left(C_{b}^{c}{\hat{C}}_{n}^{b}{\hat{u}}_{s\to d_{j}}^{n}-C_{b}^{c}\frac{r_{s\to c}^{b}}{\left|{\hat{r}}_{s\to d_{j}}^{n}\right|}\right)|}_{CDGNSS} \end{array}\right]\;H_{j}=\left[\begin{array}{ccccc} 0_{2\times 3} & 0_{2\times 3} & H_{j,\rho } & 0_{2\times 3} & 0_{2\times 3} \end{array}\right];H_{j,\rho }=\left[\begin{array}{c} \frac{\partial Az}{\partial u_{c\to d_{j}}^{c}}\\ \frac{\partial El}{\partial u_{c\to d_{j}}^{c}} \end{array}\right]C_{b}^{c}{\hat{C}}_{n}^{b}\left[{\hat{u}}_{s\to d_{j}}^{n}\times \right]\;R_{j}=\left[\begin{array}{c} \frac{\partial Az}{\partial u_{c\to d_{j}}^{c}}C_{b}^{c}{\hat{C}}_{n}^{b}\frac{\partial u_{s\to d_{j}}^{n}}{\partial r_{s\to d_{j}}^{n}}\\ \frac{\partial El}{\partial u_{c\to d_{j}}^{c}}C_{b}^{c}{\hat{C}}_{n}^{b}\frac{\partial u_{s\to d_{j}}^{n}}{\partial r_{s\to d_{j}}^{n}} \end{array}\right]{\left[\begin{array}{ccc} \sigma_{CDGNSS}\left(n\right) & 0 & 0\\ 0 & \sigma_{CDGNSS}\left(e\right) & 0\\ 0 & 0 & \sigma_{CDGNSS}\left(d\right) \end{array}\right]}^{2}{\left[\begin{array}{c} \frac{\partial Az}{\partial u_{c\to d_{j}}^{c}}C_{b}^{c}{\hat{C}}_{n}^{b}\frac{\partial u_{s\to d_{j}}^{n}}{\partial r_{s\to d_{j}}^{n}}\\ \frac{\partial El}{\partial u_{c\to d_{j}}^{c}}C_{b}^{c}{\hat{C}}_{n}^{b}\frac{\partial u_{s\to d_{j}}^{n}}{\partial r_{s\to d_{j}}^{n}} \end{array}\right]}^{T}+{\left[\begin{array}{cc} \sigma_{cam} & 0\\ 0 & \sigma_{cam} \end{array}\right]}^{2}.$$

## 5. Numerical Analysis

This section is in charge of assessing the potential of the proposed approach for bias estimation via simulation-based analyses. The necessity of a numerical approach derives from the problem dependency on the system dynamics, which makes bias estimation performance dependent not only on cooperative navigation measurements but also on the time evolution of position, velocity, and attitude, in a fully coupled fashion. Thus, a purely analytical approach such as the one proposed in [29] for positioning accuracy prediction cannot be applied. A custom camera/IMU/GNSS/magnetometer simulator has been developed for this purpose in MATLAB^®^.

Results are presented for both the cases of one and two deputies. The two-deputies case is analyzed first (Section 5.1) since in this case, full knowledge of the attitude is available and satisfying results in bias estimation are expected. In the single deputy case (Section 5.2), the attitude information is not fully available which makes some of the states unobservable, but bias estimation by cooperation can be enhanced by providing relative motion among the platforms and/or accelerated dynamics for the chief.

**Figure 3:**
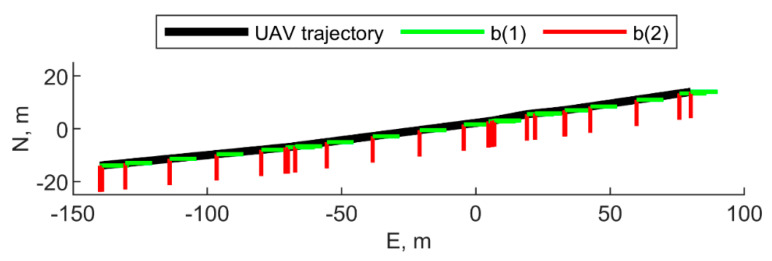
Trajectory of the chief vehicle in NED, top view. The heading angle is assumed to be equal to 90°, i.e., the vehicle is pointing eastward. The first and the second axes of the BRF are reported in the figure in green and red, respectively. UAV altitude is 20 m.

The following sub-sections assume the chief UAV moves along a quasi-straight-line trajectory, which is depicted in Figure 3. Unless differently specified, the UAV is assumed to proceed with a constant heading, with the nose pointed eastwards. To further remark the benefit of using cooperative measurements in estimating attitude, the simulated magnetometer estimate is assumed to be biased (as it actually happens in typical flight scenarios). IMU parameters used for simulating the gyroscopes and accelerometers outputs are reported in Table 1. GNSS integration uses standalone measurements, as remarked in Section 4. Results obtained by the cooperative filter are compared with those obtained when cooperative measurements are not used, i.e., the filter reported in Section 4 is used without cooperative measurements. The following sections analyze the IMU biases estimation performance.

### 5.1. Two Deputies

**Figure 4:**
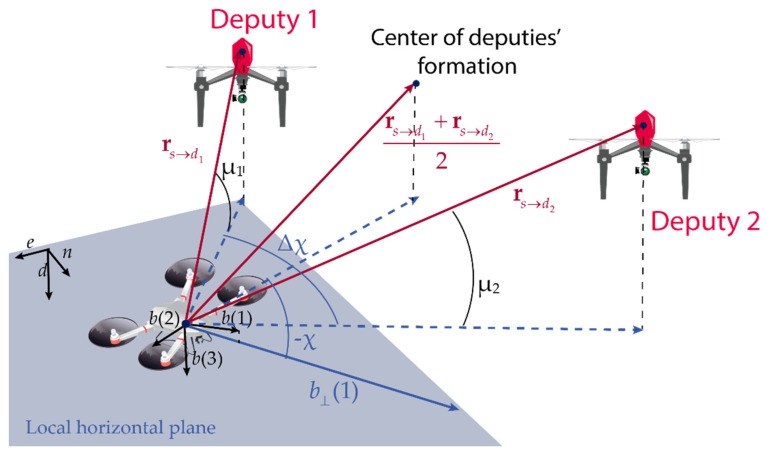
Formation geometry of a triplet of vehicles composed by one chief and two deputies. The local horizontal plane is depicted in blue. Projections on that plane are indicated with blue lines.

When two deputies are used, the full attitude information can be estimated if chief and the two deputies are not aligned with each other. Figure 4 shows the formation geometry, which is defined by the elevation of each deputy with respect to the chief, i.e., μ*_j_*, its range (*r*_*j*_), the separation between the two deputies on the local horizontal plane (Δχ), and the angle between the projection of chief’s forward direction on the local horizontal plane, i.e., $b_{⊥}(1)$, and the center of deputy formation, i.e., χ, which is positive if defined clockwise along the down direction.

Depending on the geometry, cooperative navigation can be less or more accurate in terms of estimation of different attitude angles, which influences the process of bias estimation, especially concerning the accelerometers. Reference [35] demonstrates that using a formation of two UAVs centered along the UAV forward direction, i.e., roll axis (χ = 0°), cooperative aiding is more effective on pitch estimate if the horizontal angle between the two deputies (Δχ) is smaller. On the contrary, when Δχ increases, the roll angle is characterized by a better accuracy. By posing χ = 90°, the behavior inverts, giving a more accurate roll estimate with small Δχ. In this section, the influence of the triplet’s formation geometry is analyzed by posing *r*_1_ = *r*_2_ = *r*, μ_1_ = μ_2_ = μ, and χ = 0, and varying μ and Δχ.

**Figure 5:**
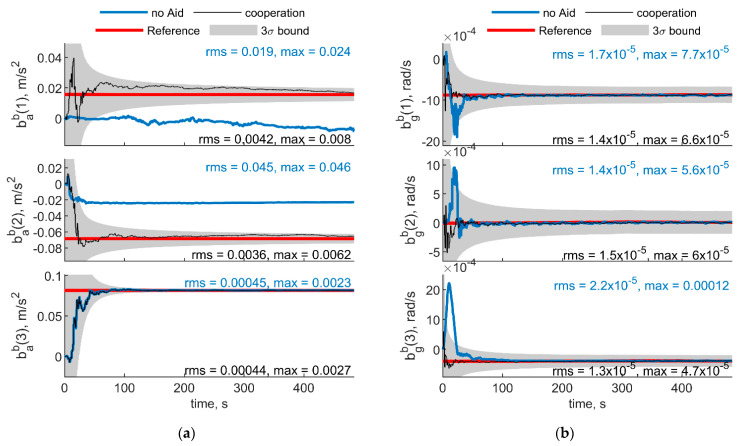
(**a**) Accelerometer and (**b**) gyroscope biases predicted by the filter. Chief trajectory is reported in Figure 3. Two deputies, *r* = 100 m, μ = 0°, Δχ = 70°. Reference value is reported in red. Results obtained with cooperation and without cooperation have been reported in black and blue, respectively. RMS and maximum error value have been evaluated starting from t = 60 s.

**Figure 6:**
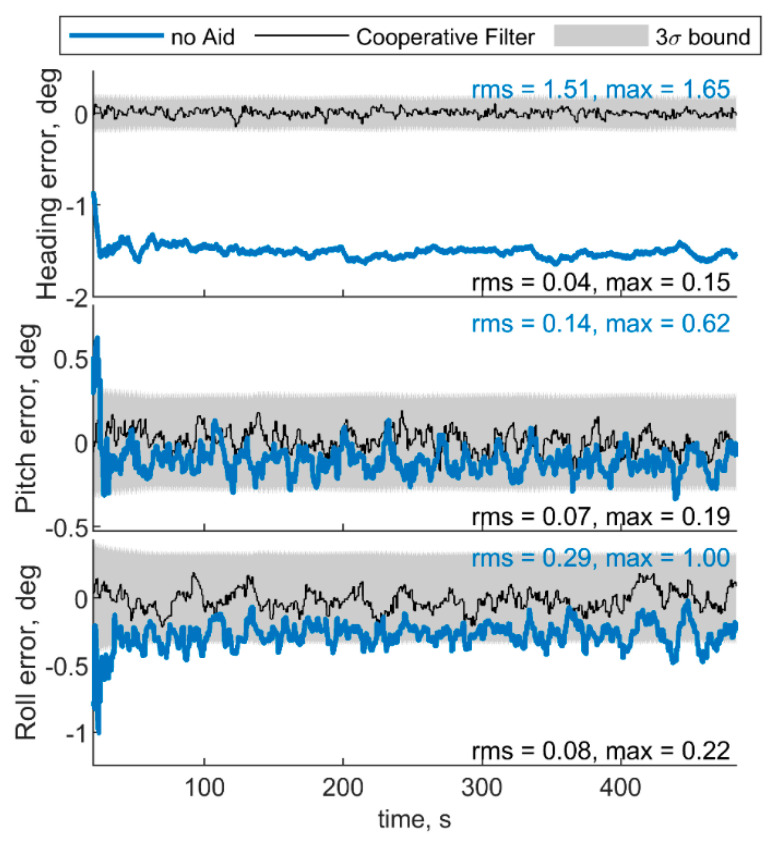
Errors on angle estimated by the cooperative filter in blue and by the same filter without using cooperation. Chief trajectory is in reported in Figure 3. Two deputies, *r* = 100 m, μ = 0°, Δχ = 70°.

Figure 5 and Figure 6 report the results in the case the trajectory depicted in Figure 3 has been assumed for the chief vehicle, while the relative deputies’ geometry is given by μ = 0°, Δχ = 70°, *r* = 100 m. Figure 5a,b shows the accelerometer and gyroscope biases estimated by the cooperative filter (in black) compared with those estimated without cooperation (blue) and with the simulated biases, i.e., reference solution, in red. The 3σ bound derived by estimating the error STD (i.e., σ) with the filter predicted covariance, is also reported in gray. Root mean square (RMS) and maximum errors are reported for cooperation-aided and non-cooperative filter, removing the first 60 s needed for filter convergence. As concerns gyroscopes, cooperative navigation measurements allow the filter to converge to the true bias values faster, due to more accurate heading estimate provided by CDGNSS/vision measurements compared with magnetometer. As far as accelerometers’ biases are concerned, the estimate is dramatically improved using cooperation. In fact, cooperative measurements allow convergence to the reference value, which otherwise would not be achieved.

For the sake of completeness, Figure 6 shows the attitude errors, remarking the effectiveness of cooperation especially in heading estimate, which is debiased due to the IMU/magnetometer independent nature of the CDGNSS/vision measurement. RMS and max errors are reported both for cooperative filter and for the filter which does not use cooperative measurements.

**Figure 7:**
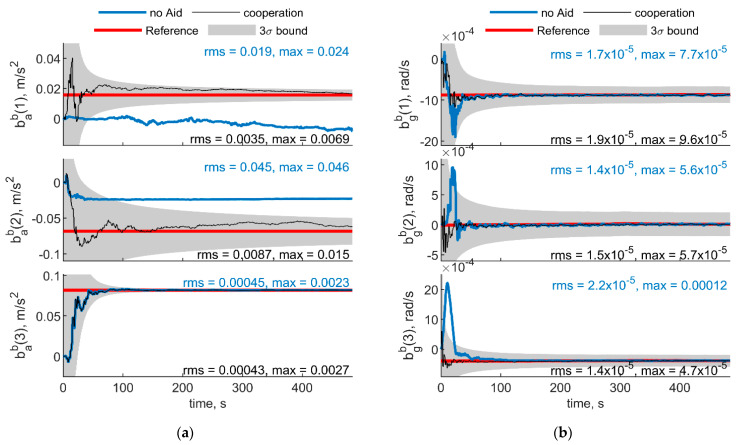
(**a**) Accelerometer and (**b**) gyroscope biases predicted by the filter. Chief trajectory is reported in Figure 3. Two deputies, *r* = 100 m, μ = 0°, Δχ = 20°. Reference value is reported in red. Results obtained with cooperation and without cooperation have been reported in black and blue, respectively. RMS and maximum error value have been evaluated starting from t = 60 s.

**Figure 8:**
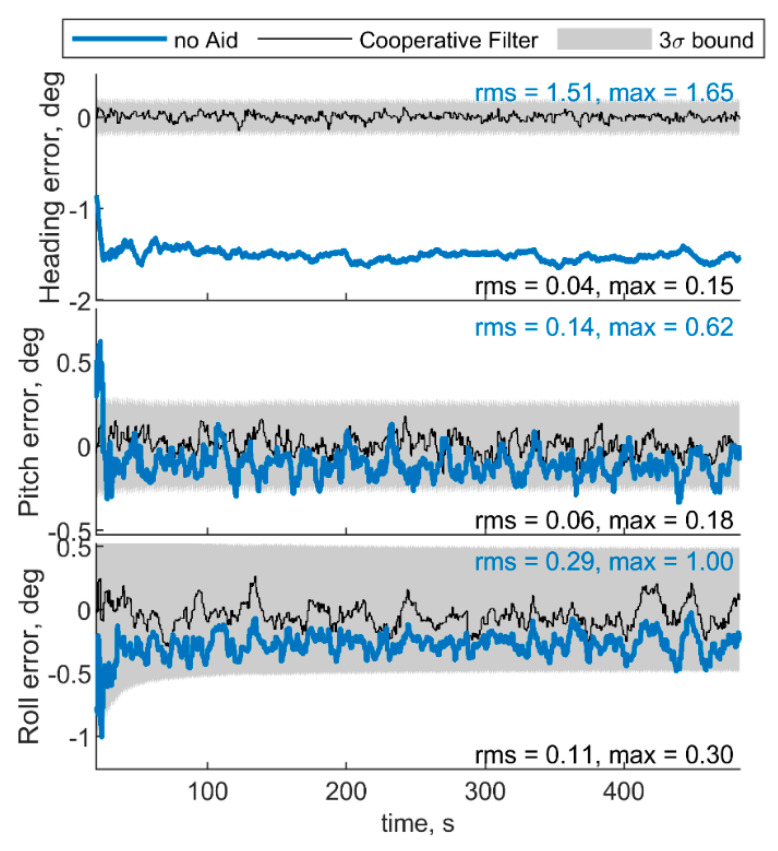
Errors on angle estimated by the cooperative filter in blue and by the same filter without using cooperation. Chief trajectory is in reported in Figure 3. Two deputies, *r* = 100 m, μ = 0°, Δχ = 20°.

Figure 7 and Figure 8 show respectively IMU biases and angular errors resulting when the angular separation between deputies (i.e., Δχ) is reduced from 70° to 20°. The other parameters have been assumed equal to the previous case. With respect to the previous analyzed case (Figure 5 and Figure 6), it could be seen that pitch error slightly reduces with an increase of roll error as predicted by [35]. Whereas bias acceleration RMS decreases on the first axis and increases along the second axis of the BRF. This behavior can be justified by referring to the derivations of [11]. Reference [11] explicitly derives the connection between the north angle error and east accelerometer bias, and between the east angle error and the north accelerometer biases, grouping the two couples in two of the six unobservable subsets in ZUPT calibration. Without loss of generality, for a quasi-leveled flight with small pitch and roll angle (which holds true in quadrotor flight avoiding aggressive flight conditions), one can extend the dependencies found in [11] in BRF. Using the rotation matrix, one can find two couples (i.e., subsets) of linear dependent errors: pitch error and accelerometer bias along the first axis of the BRF, i.e., $b_{a}^{b}\left(1\right)$, and roll error and accelerometer bias along the second axis of the BRF, i.e., $b_{a}^{b}\left(2\right)$. Therefore, any attempt to improve pitch accuracy (e.g., reducing Δχ from 70° to 20°) will reduce the error of one of the elements of the first subset. Indeed, comparison between Figure 5 and Figure 7 shows a reduction of $b_{a}^{b}\left(1\right)$, and an increase of $b_{a}^{b}\left(2\right)$ RMS values. This is further highlighted by the increased value of the covariance of $b_{a}^{b}\left(2\right)$, with respect to the previous case. Conversely, gyroscope bias slightly varies with respect to the Figure 5 case. Indeed, horizontal gyroscopes’ biases mostly depend on position covariance, while the bias of the gyroscope along the third axis is proportional to the heading error [11].

**Figure 9:**
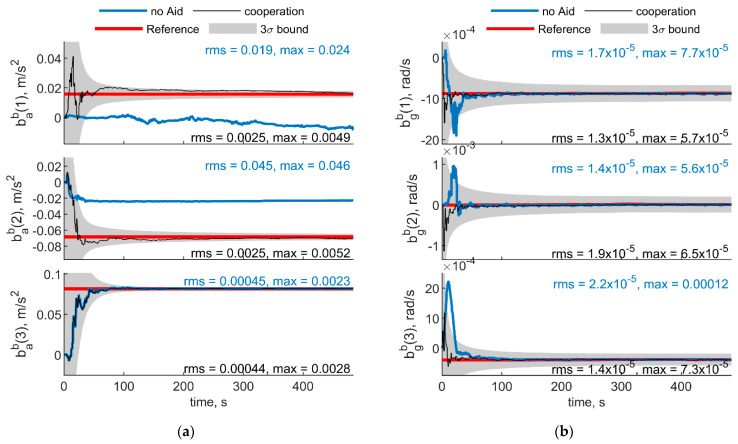
(**a**) Accelerometer and (**b**) gyroscope biases predicted by the filter. Chief trajectory is reported in Figure 3. Two deputies, *r* = 100 m, μ = 60°, Δχ = 70°. Reference value is reported in red. Results obtained with cooperation and without cooperation have been reported in black and blue, respectively. RMS and maximum error value have been evaluated starting from t = 60 s.

**Figure 10:**
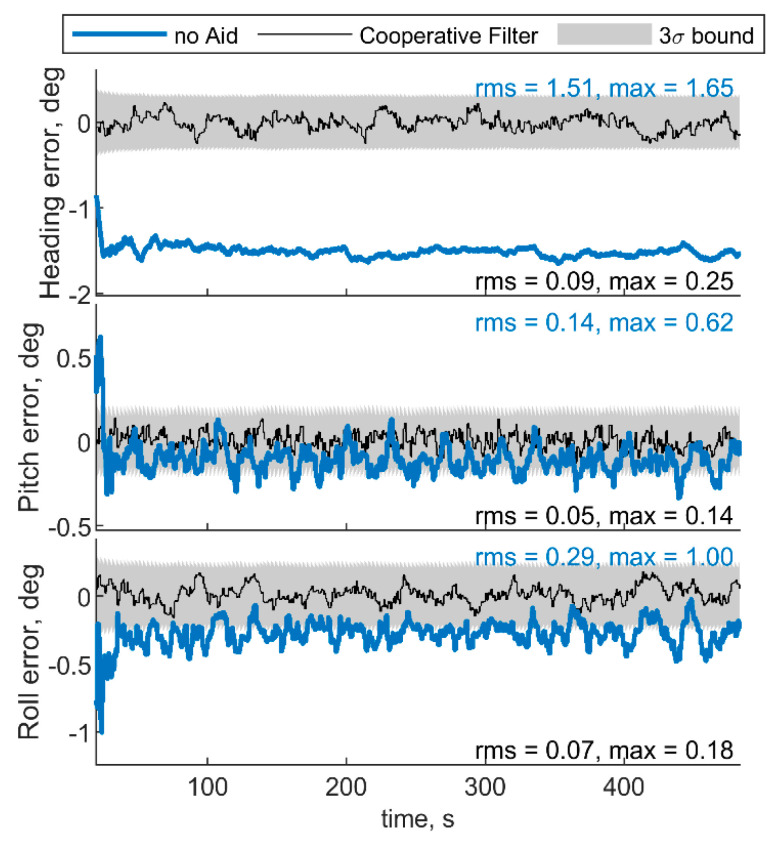
Errors on angle estimated by the cooperative filter in blue and by the same filter without using cooperation. Chief trajectory is reported in Figure 3. Two deputies, *r* = 40 m, μ = 60°, Δχ = 70°.

When μ increases up to 90°, cooperative measurements have more impact on the horizontal plane angles (pitch and roll) than on heading. Results obtained using Δχ = 70° and increasing μ up to 60°, are reported in Figure 9 and Figure 10 for IMU biases and angular errors, respectively. 

Comparing these results with those reported in Figure 5 and Figure 6 (i.e., with the same value of Δχ, but with a null μ), one can notice an increase of heading RMS and covariance, with a reduction on the horizontal angles covariances and RMS. Conversely, an improvement in horizontal accelerometers’ biases estimation is provided. Additionally, down gyroscope bias estimation error, i.e., $b_{g}^{b}\left(3\right)$, slightly increases, due to the increase of heading error and its covariance.

To highlight the benefit of having a large baseline, Table 2 compares the results obtained with the same formation geometries described before, but with a distance between deputies and chief, i.e., *r*, reduced from 100 to 40 m.

### 5.2. One Deputy

The relative geometry between the chief and the single deputy, (if constant with time) can be defined by referring to Figure 4, with Δχ = 0. In this case, the position of the deputy UAV coincides with the center of the formation and only χ, μ, and *r* are needed to uniquely identify the relative formation geometry.

**Figure 11:**
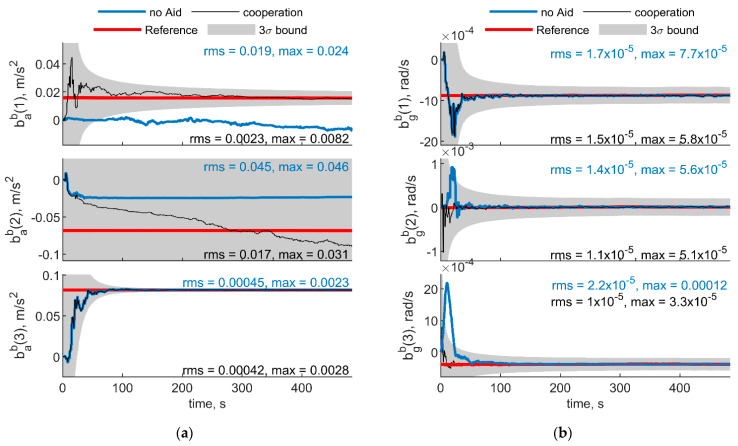
(**a**) Accelerometer and (**b**) gyroscope biases predicted by the filter. Chief trajectory is reported in Figure 3. One deputy, *r* = 100 m, μ = 0°, χ = 0°. Reference value is reported in red. Results obtained with cooperation and without cooperation have been reported in black and blue, respectively. RMS and maximum error value have been evaluated starting from t = 60 s.

**Figure 12:**
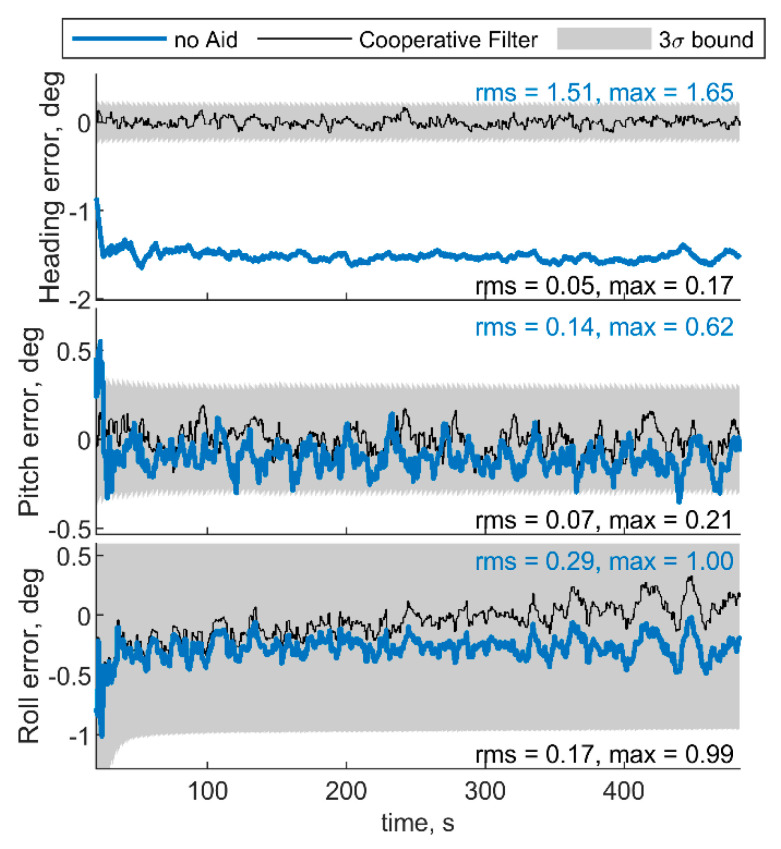
Errors on angle estimated by the cooperative filter in blue and by the same filter without using cooperation. Chief trajectory is reported in Figure 3. One deputy, *r* = 100 m, μ = 0°, Δχ = 0°.

When only one deputy is available, the measurements provided to the filter, i.e., CDGNSS/vision residuals for attitude, and GNSS observables for position, do not give enough information to ensure full observability of the filter state. As an example, the results obtained by routing the chief UAV along the trajectory reported in Figure 3, and assuming a deputy with a fixed relative geometry with χ = 0°, μ = 0°, and *r* = 100 m (i.e., deputy along the roll direction) are shown in Figure 11 and Figure 12, for IMU biases and angular error respectively. Since cooperative aiding is effective only in the directions orthogonal to the LOS, roll angle error increases due to unobservability as well as the error on the accelerometer bias of the second axis, as a consequence of the dependence among these two variables, demonstrated in [11]. However, both the unobservable variables are well within their 3σ bound.

To cope with these observability challenges in the case of a single deputy, different strategies can be proposed. In particular, relative geometry variation and accelerated chief motion are analyzed in the following. Section 5.2.1 reports the result with improved chief dynamics. Whereas Section 5.2.2 reports the results obtained by making the relative geometry change when the chief is routed along a straight line.

#### 5.2.1. Accelerated Dynamics of the Chief Vehicle

**Figure 13:**
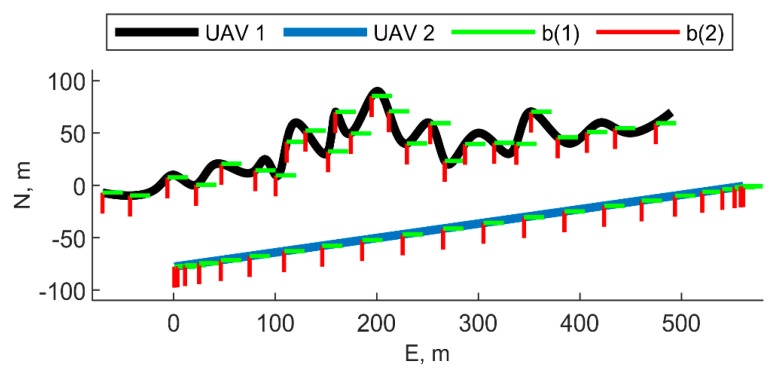
Trajectory of the chief and deputy vehicles, top view. For both the vehicles, the heading angle is assumed to be equal to 90°, i.e., the vehicles are pointing eastward. The first and the second axes of the BRFs are reported in the figure, whereas the third axis is pointing downward. UAVs altitude is 20 m.

**Figure 14:**
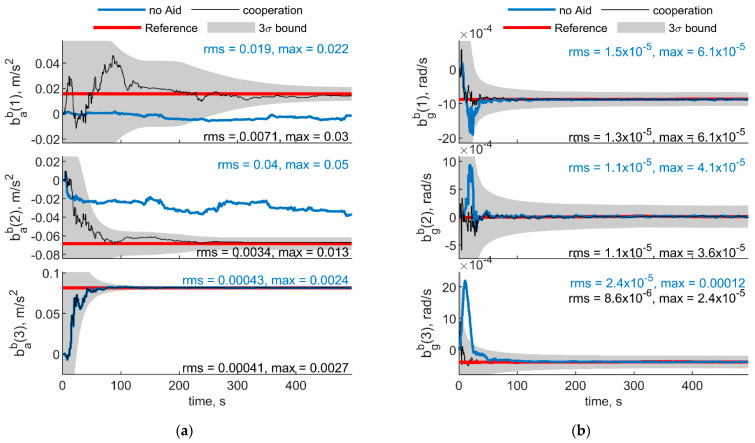
(**a**) Accelerometer and (**b**) gyroscope biases predicted by the filter. One deputy. Chief and deputy move along UAV 1 and UAV 2 trajectories reported in Figure 13, respectively. Reference value is reported in red. Results obtained with cooperation and without cooperation have been reported in black and blue, respectively. RMS and maximum error value have been evaluated starting from t = 60 s.

**Figure 15:**
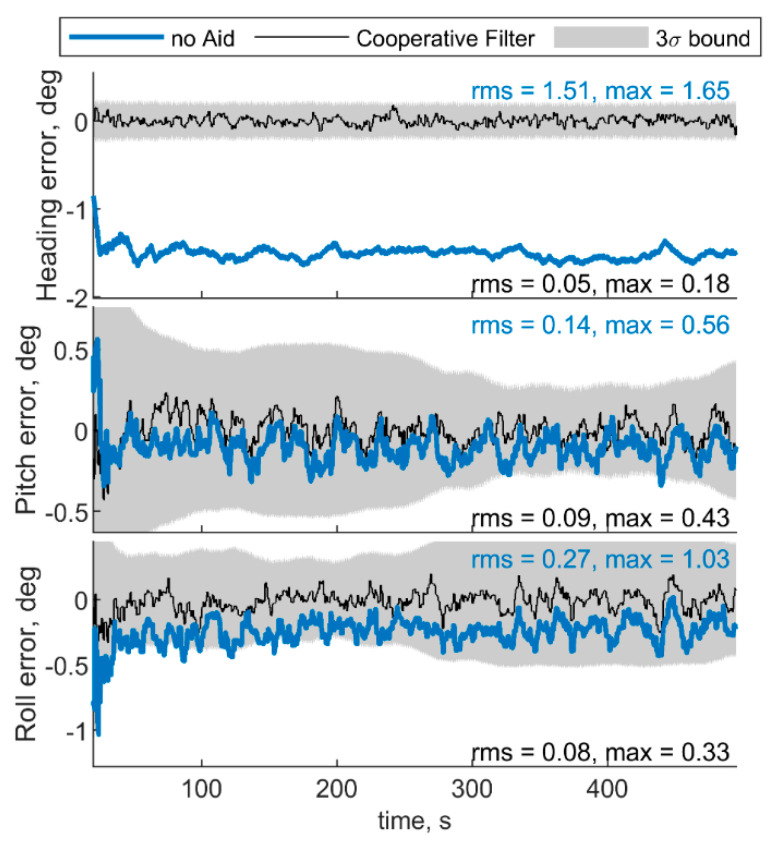
Errors on angle estimated by the cooperative filter in blue and by the same filter without using cooperation. One deputy. Chief and deputy move along UAV 1 and UAV 2 trajectories reported in Figure 13, respectively.

In this section, it is assumed the chief moves along a zig-zag path, whose top view is depicted in Figure 13. The chief (UAV 1 in the figure) is always pointed toward east, with a 90° heading angle. The trajectory of the deputy (UAV 2) is also depicted in the figure. The deputy moves along a straight line with a 90° heading angle. The two vehicles fly at constant altitude equal to 20 m. Cooperative filter results in terms of IMU biases and angular errors are reported in Figure 14 and Figure 15. 

Choosing a zero elevation (vehicles flying at the same altitude) allows the cooperative measurements to give a significant contribution to heading angle estimation. Indeed, due to the small heading angle covariance of CDGNSS/vision, magnetometer measurements are filtered out by the filter, allowing the heading estimate to be debiased. As far as accelerometer biases are concerned, after the initial excursion, which holds true for the accelerometer bias on the first component, the relative geometry variation and the chief dynamics improve the state observability, providing a very accurate estimation in accelerometer biases.

#### 5.2.2. Relative Geometry Variation

Relative geometry variation allows the LOS direction to change during the motion, which introduces spatial diversity in the measurements and is useful to tackle the observability challenges which characterize a constant relative geometry. Three different relative geometries have been taken into account in this section, assuming the chief vehicle is always flown along a straight line.

1. Chief and deputy vehicles move along the trajectories of UAV 2 and UAV 1 reported in Figure 13, respectively (i.e., they are inverted with respect to the Section 5.2.1);
2. Chief flies along the quasi-straight-line path reported in Figure 3 by continuously rotating its heading with a 100 s period, starting from an initial heading angle, ψ = 90°. The deputy moves along a trajectory parallel to the chief, which has been defined with r = 100, μ = 12°, and χ = 30° in the initial point of the trajectory;
3. Chief flies along the quasi-straight-line path reported in Figure 3 with a constant heading angle assumed equal to 90°. The deputy is steady and its NED position vector is [−100 m, −20 m, −40 m]*^T^*.

Cases 2 and 3 assume a large camera FOV, which can be achieved with omnidirectional [38] or multiple cameras system mounted on the chief platforms. For the sake of brevity, only bias results are reported in the following subsections, whereas angular RMS errors are indicated in the text.

**Figure 16:**
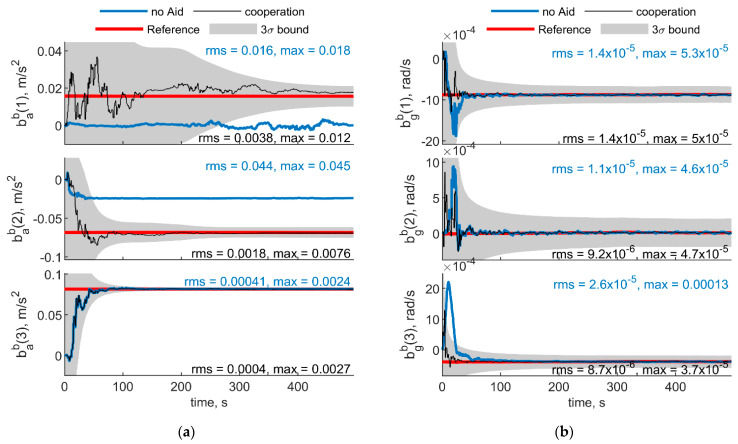
(**a**) Accelerometer and (**b**) gyroscope biases predicted by the filter. One deputy. Chief and deputy move along UAV 2 and UAV 1 trajectories reported in Figure 13, respectively, as reported in case 1. Reference value is reported in red. Results obtained with cooperation and without cooperation have been reported in black and blue, respectively. RMS and maximum error value have been evaluated starting from t = 60 s.

Results of case 1 are reported in Figure 16. Angular RMS errors are 0.05, 0.08, and 0.08 degrees for heading, pitch and roll angles, respectively. Accelerometer bias estimation overperforms the one obtained in the case the two UAVs invert their trajectory (presented in the previous section and reported in Figure 14), demonstrating that more that ownership dynamics, relative geometry variation plays a significant role in cooperative bias estimation. First axis bias still presents huge excursions in the first epochs, before the convergence is encountered when a satisfactory set of measurements have been acquired in terms of spatial diversity.

**Figure 17:**
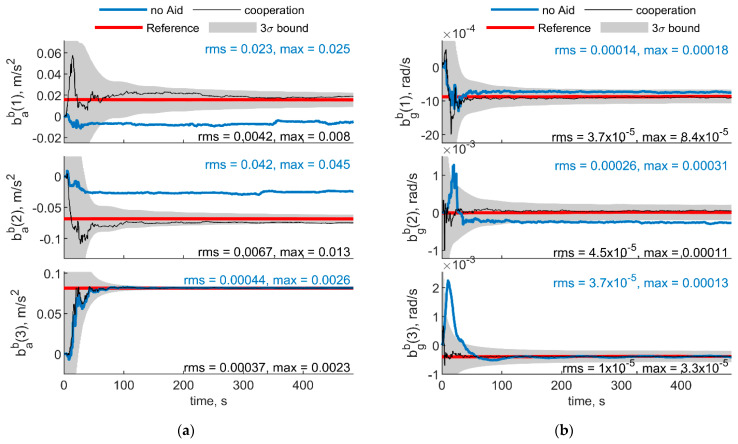
(**a**) Accelerometer and (**b**) gyroscope biases predicted by the filter. One deputy. Chief and deputy formation identified by case 2. Reference value is reported in red. Results obtained with cooperation and without cooperation have been reported in black and blue, respectively. RMS and maximum error value have been evaluated starting from t = 60 s.

Result obtained by changing the heading of the chief, i.e., case 2, are reported in Figure 17. Differently from the other cases, heading rotation negatively impacts the gyroscope bias estimation along the horizontal axes if no cooperative measurements are provided. On the other hand, using cooperation allows both accelerometer and gyroscopes measurements to be debiased. Attitude RMS error obtained using cooperation are 0.06, 0.08, and 0.09 for heading pitch and roll, respectively.

**Figure 18:**
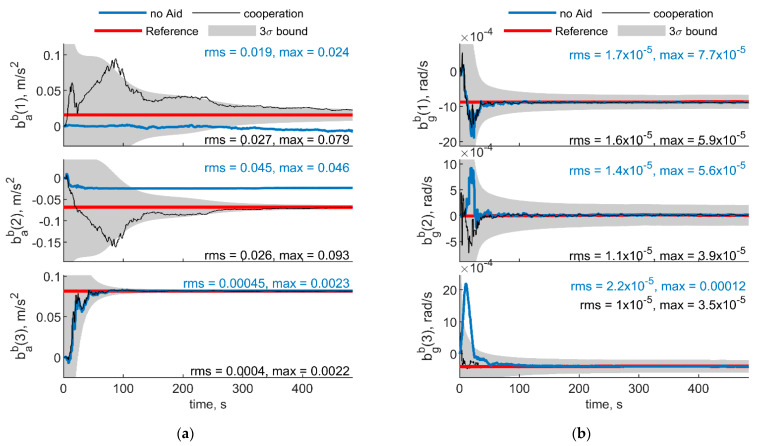
(**a**) Accelerometer and (**b**) gyroscope biases predicted by the filter. One deputy. Chief and deputy formation identified by case 3. Reference value is reported in red. Results obtained with cooperation and without cooperation have been reported in black and blue, respectively. RMS and maximum error value have been evaluated starting from t = 60 s.

As concerns case 3, observability of the full state has been performed by providing spatial diversity while making the chief UAV fly along the trajectory reported in Figure 3 and assuming a steady deputy UAV. This scenario can also model the case in which ground GNSS antennas are used as fixed deputies. Results are reported in Figure 18. The formation geometry provides the least advantage with respect to the solutions presented before, because enough spatial diversity in the measurements is obtained after a long time (i.e., 200 s). At that time, the relative azimuth variation between the chief and the deputy vehicle is about 30°, which provide sufficient spatial diversity to make the biases converge. Before this time interval, the results present a very inaccurate accelerometers’ bias estimation. However RMS value reduces to [13.0 7.6 0.11] × 10^−3^ m/s^2^ if estimated after this time interval.

**Figure 19:**
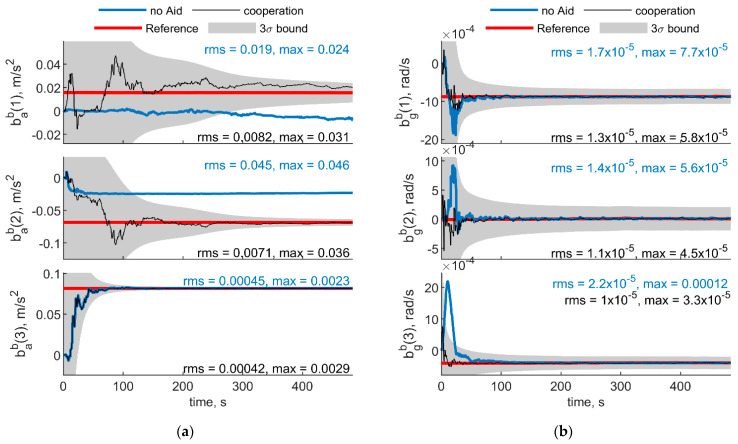
(**a)** Accelerometer and (**b**) gyroscope biases predicted by the filter. One deputy. Chief flies along the trajectory depicted in Figure 3 and deputy is steady at [−100 m, −20 m, −20 m]*^T^* in NED coordinates. Reference value is reported in red. Results obtained with cooperation and without cooperation have been reported in black and blue, respectively. RMS and maximum error value have been evaluated starting from t = 60 s.

A solution which allows improving the performance in bias prediction when a steady deputy is used consists of providing a null elevation between the chief and the deputy so that the heading direction (which is the most inaccurate since it is based on biased magnetometer estimates) is always observable with cooperative measurements. Figure 19 shows the result obtained with a steady deputy with the same horizontal position of case 3 (Figure 18), but with the same altitude of the chief vehicle. So that deputy NED position is [−100 m, −20 m, −20 m]*^T^*.

## 6. Experimental Set-Up and Results

**Figure 20:**
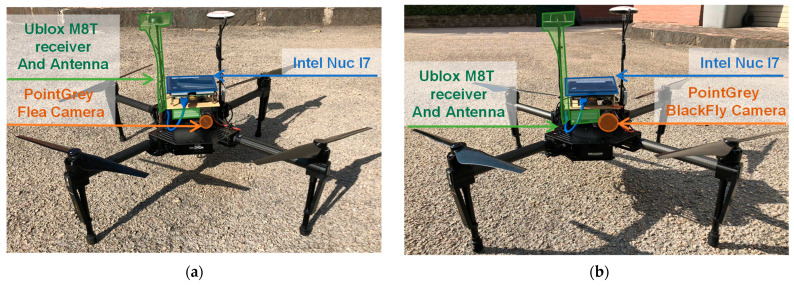
(**a**) Eagle and (**b**) Athena setup.

The efficiency of the proposed method for IMU bias estimation has been tested on experimental data, acquired at a model aircraft airfield. The data acquisition setup is composed by two DJI^TM^ M100 drones and a Trimble antenna. The flight has been carried out by remotely piloting the two drones, which are shown in Figure 20. The drones, named Eagle and Athena, have been equipped each with an onboard computer, a CCD camera and an additional GNSS receiver with raw data capability. The latter is required due to the impossibility of reading GNSS raw data directly from DJI autopilot telemetry.

- Eagle has as onboard computer, an Intel NUC^TM^ with an i7 CPU running Ubuntu 14.04. It embarks a PointGrey FleaTM FL3-U3-20E4C-C CCD camera (with 1600 × 1200 resolution in pixels, maximum frame rate of 59 fps and an IFOV of about 0.030°) and a uBlox^TM^ LEA-M8T GNSS single frequency multi-constellation receiver with raw measurements capabilities.
- Athena has been equipped with an onboard computer (Intel NUC^TM^ with an i5 CPU running Ubuntu 16.04), a CCD camera, i.e., PointGrey Blackfly^TM^ BFLY-U3-50H5C-C (with 2448 × 2048 resolution in pixels, maximum frame rate of 7.5 fps, and an IFOV of about 0.022°) and the same GNSS receiver with raw data capability embarked on Eagle, i.e., uBlox^TM^ LEA-M8T.

uBlox^TM^ receivers have been set with both GPS and Galileo receiver capability. Whereas only GPS data were available at Trimble ground antenna. As Figure 20 shows, the uBlox^TM^ antenna has been mounted symmetrically to the DJI one, on each drone. Both the DJI and uBlox antenna have been placed on a carbon fiber rod higher than the DJI default, to avoid possible interference with the onboard computer.

Data acquisition software capable of retrieving DJI autopilot and IMU, camera and raw GNSS data have been developed in ROS (robot operating system). Using ROS allows easily time-tagging and synchronizing acquired data using custom and already developed (DJI^TM^ and Pointgrey^TM^ proprietary) ROS nodes. A custom made node was developed in C++ to acquire uBlox^TM^ raw data [39] in user readable format. Camera calibration has been performed indoor using the Kalibr software [40].

The data acquired during the flight campaign have been processed offline within a MATLAB^®^ implementation of the cooperative navigation filter reported in Section 4, assuming Eagle as the chief vehicle and the two deputies being Athena and the Trimble antenna. Accurate 3D positions of GNSS satellites have been calculated using the multi-constellation broadcast ephemeris file in a customized version of the RTKLIB software [41], able to provide multi-constellation satellite positions and pseudoranges corrected from ionospheric and tropospheric errors. CDGNSS baselines have been retrieved by the “kinematic” mode of the RTKLIB software [41], using GNSS raw data acquired on board the chief and the two deputies. As concerns camera information, several techniques have been developed by the authors in the framework of cooperative detection, e.g., using deep learning [42]. This strategy complemented with a supervised approach has been used to acquire camera data, i.e., pixels of the deputies’ center, in this paper, being the focus set on the cooperative filter. Camera and CDGNSS STDs have been retrieved from camera specifics (i.e., IFOV) and from resulting STD of RTKLIB “kinematic solution”, respectively. IMU parameters needed to define the process noise covariance matrix, i.e., velocity and angular random walk and gyroscope and accelerometer bias instabilities have been derived thanks to IMU calibration based on Allan variance analysis performed with Kalibr software [40].

**Figure 21:**
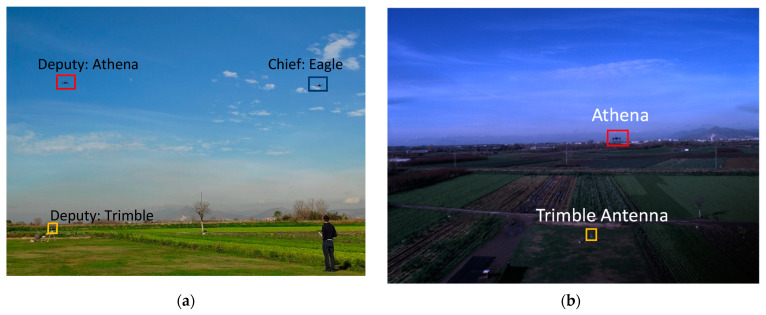
(**a**) Flight image, (**b**) Image acquired by chief vehicle (Eagle) during the flight.

Figure 21a shows an image taken during the flight where the three platforms (two deputies and one chief) are highlighted. A flight image taken by the chief vehicle including both the deputies is reported in Figure 21b. Both one deputy and two deputies cases are analyzed.

**Figure 22:**
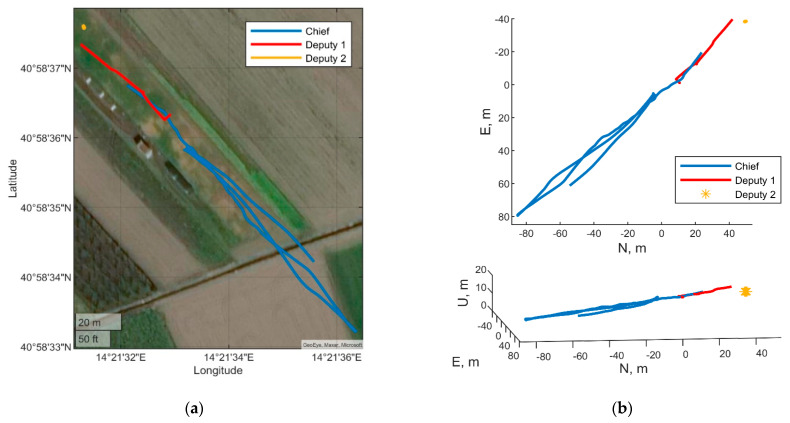
Path of the chief and the two deputies. Two deputies case: (**a**) Longitude, latitude coordinates, (**b**) ENU coordinates top view and 3D view.

**Figure 23:**
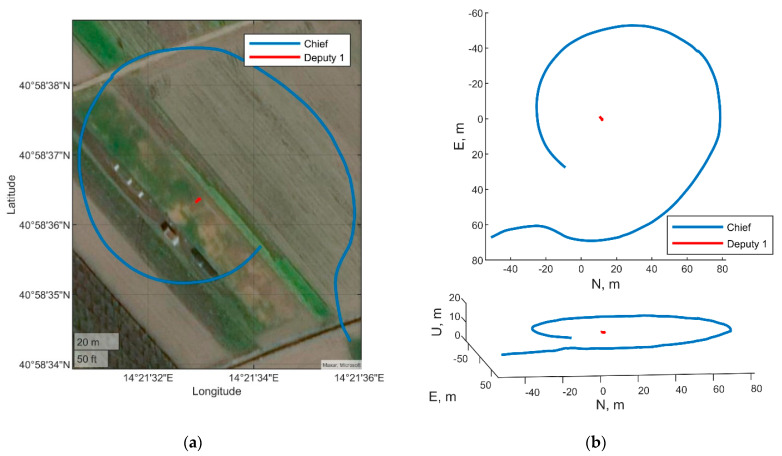
Path of the chief and the deputy. One deputy case: (**a**) Longitude, latitude coordinates, (**b**) ENU coordinates top view and 3D view.

- The two deputies case uses Eagle as chief UAV, a flying deputy (Athena UAV) and a ground antenna (Trimble) as surrogate deputy. The paths of the three “vehicles”, estimated by uBlox receivers for the two drones and by RTKLIB processing for the Trimble antenna are reported in Figure 22. Figure 22a shows the latitude longitude coordinates of the paths reported on a satellite image. Whilst east north up (ENU) coordinates are reported in Figure 22b, where top and 3D views are reported. These paths are relevant to a limited segment (from 334 to 449 s) of the entire dataset acquired during the flight campaign, where both the deputies are within the field of view of the chief’s camera.
- The one deputy case uses Eagle as chief vehicle and Athena as deputy vehicle, exploiting proper dynamics of the two platforms. Specifically, Athena holds an almost steady position whilst Eagle is rotating around it and changing its heading with the aim of always keeping the deputy in its camera FOV. The horizontal acceleration of the chief and the variation of chief-deputy LOS in BRF both provide benefits to inertial bias observability. Figure 23 reports the trajectory of the two vehicles in latitude-longitude coordinates (Figure 23a) and in top and 3D view (Figure 23b). Eagle performs a circle around Athena in the time epoch going from 476 to 551 s of the acquired dataset.

**Figure 24:**
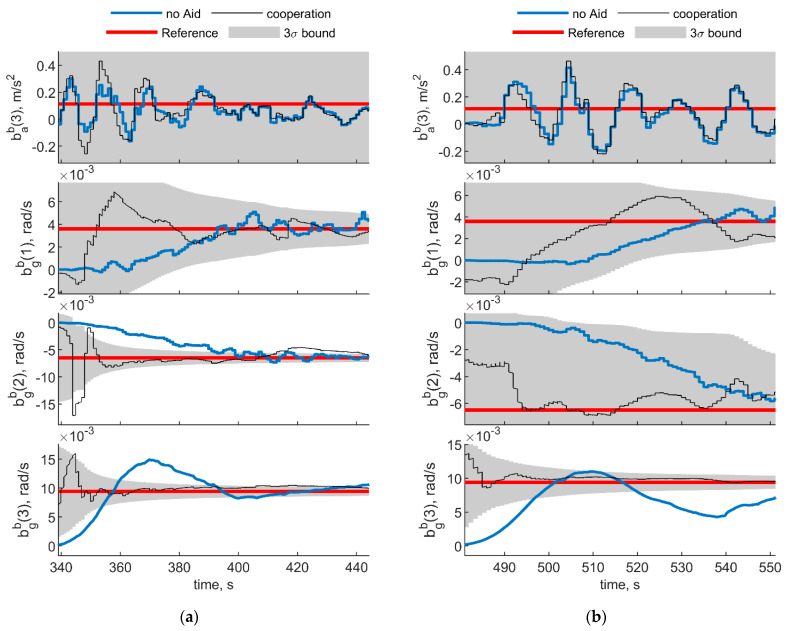
Down accelerometer and gyroscopes biases experimental results. (**a**) Two deputies. Chief and deputy trajectories are reported in Figure 22. (**b**) One deputy. Chief and deputy trajectories are reported in Figure 23. Reference value obtained from the ZUPT filter is reported in red, whereas results obtained with cooperation and without cooperation have been reported in black and blue, respectively. 3σ bound is reported in gray.

To have a reference for accelerometer and biases estimated quantities, a ZUPT filter has been used for the first 70 s of the test, where Eagle platform has been kept in static conditions. The ZUPT filter uses inertial equations for propagation and correct the state by informing the filter a zero velocity is experienced. To guarantee observability of the third component of the gyroscope bias and heading angle, the ZUPT filter used in this paper also uses the magnetometer measurement in correction step. However, both $b_{a}^{b}\left(1\right)$ and $b_{a}^{b}\left(2\right)$ are unobservable from the ZUPT filter, because their estimated covariance is far higher than the estimated value for the biases, and cannot be used as a benchmark to evaluate the effectiveness of cooperation. Therefore, only benchmarked values (i.e., the three gyroscope and the down accelerometer biases) are reported in Figure 24. Figure 24 shows the IMU biases estimated with the navigation filter reported in Section 4. Figure 24a,b depicts the results obtained considering trajectories reported in Figure 22 (two deputies case) and Figure 23 (one deputy case), respectively. As in the previous section, results with and without cooperation have been reported by black and blue lines. Reference values, obtained as the values to which the ZUPT filter converges, are also reported in red, and enclosed within the gray 3σ bound.

In the two deputies case, the chief UAV is always looking at the deputies and both the multirotors exhibit a limited motion. This results in a scenario with constant geometry and variable range, with the two deputies having a very small Δχ and a χ near to 0°. In the single deputy case, the deputy is always within the chief FOV, providing a relative azimuth variation of about 40°.

In both cases, cooperative estimation allows rapidly estimating gyroscope biases and yields a significant advantage with respect to the non cooperative filter especially on the down axis gyroscope bias estimation, which would otherwise be negatively impacted by the wrong magnetometer estimation. Down accelerometer bias oscillates around the true value within the covariance bound both for cooperative and uncooperative results.

## 7. Conclusions

This paper analyzed the potential of a cooperative navigation strategy based on one or more deputy aircraft for improved in-flight estimation of inertial sensors biases. Combination of relative LOS measurements provided by camera(s) and CDGNSS measurements provides inertial-independent attitude information which can be exploited for bias estimation. A numerical analysis shows that using two deputies give a full observable state for the proposed navigation filter. However, relative formation geometry affects the biases estimation process and observability can be maximized by properly tailoring deputies’ trajectories. Distance between chief and deputy platforms plays a significant role and provides more accurate estimates when increased, if visual measurements can be still extracted with pixel-level uncertainty. When a single deputy is available, full observability is not guaranteed which can be tackled by different strategies. In particular, continuously varying the relative geometry between the chief and the deputy provides spatial diversity of the measurements and improves observability. When magnetic sensors are used, the negative effects of magnetometer biases can be effectively counteracted by keeping low elevation angles and thus maximizing heading observability by cooperative measurements. First experimental results, obtained in the case of one and two deputies, also demonstrate that the proposed methodology can improve accuracy in in-flight inertial bias estimation. Future research is aimed at further demonstrating the concept in flight high performance inertial units within an ad hoc extensive flight campaign. This will also allow a deeper analysis of the effects of non idealities that are found in experimental conditions.

## Figures and Tables

**Table 1:** IMU parameters [37].

Velocity RW ^1^ [m/s/√h]	Acc. Bias Stability [mg]	Acc. Bias Repeatability [mg]	Angular RW [°/√h]	Gyro Bias Stability [°/h]	Gyro Bias Repeatability [°/h]
0.04	0.03	5	0.3	10	260

^1^ RW is the random walk.

**Table 2:** RMS and maximum error by varying range of the formation. Only cooperative filter results RMS and maximum error are reported.

Angular Geometry	Range [m]	Acc. Bias Errors [m/s^2^]	Gyro Bias Errors [m/s^2^]	Angle Errors (ψ, θ, φ) [deg]
μ = 0°, Δχ = 20°	100	RMS [3.5, 8.7, 0.43] × 10^−3^ Max [6.9, 15, 2.7] × 10^−3^	RMS [1.9, 1.5, 1.4] × 10^−5^ Max [9.6, 5.7, 4.7] × 10^−5^	RMS [0.04, 0.06, 0.11] Max [0.15, 0.18, 0.30]
	40	RMS [6.5, 9.1, 0.42] × 10^−3^ Max [12.0, 20, 2.6] × 10^−3^	RMS [1.8, 1.5, 1.4] × 10^−5^ Max [9.2, 6.1, 5.4] × 10^−5^	RMS [0.05, 0.09, 0.10] Max [0.18, 0.25, 0.38]
μ = 0°, Δχ = 70°	100	RMS [4.2, 3.6, 0.44] × 10^−3^ Max [8.0, 6.2, 2.7] × 10^−3^	RMS [1.4, 1.5, 1.3] × 10^−5^ Max [6.6, 6.0, 4.7] × 10^−5^	RMS [0.04, 0.07, 0.08] Max [0.15, 0.19, 0.22]
	40	RMS [8.6, 7.6, 0.43] × 10^−3^ Max [14.0, 12.0, 2.7] × 10^−3^	RMS [2.0, 1.5, 1.3] × 10^−5^ Max [10.0, 5.9, 4.6] × 10^−5^	RMS [0.05, 0.10, 0.10] Max [0.16, 0.28, 0.28]
μ = 60°, Δχ = 70°	100	RMS [2.5, 2.5, 0.44] × 10^−3^ Max [4.9, 5.2, 2.8] × 10^−3^	RMS [1.3, 1.9, 1.4] × 10^−5^ Max [5.7, 6.5, 7.3] × 10^−5^	RMS [0.09, 0.05, 0.07] Max [0.25, 0.14, 0.18]
	40	RMS [6.1, 4.2, 0.44] × 10^−3^ Max [9.6, 7.3, 2.8] × 10^−3^	RMS [2.7, 1.8, 3.4] × 10^−5^ Max [12.0, 7.1, 17.0] × 10^−5^	RMS [0.14, 0.08, 0.09] Max [0.44, 0.22, 0.27]

## Data Availability

Data sharing is not applicable to this article.
